# Upregulation of Nrf2/HO-1 Signaling and Attenuation of Oxidative Stress, Inflammation, and Cell Death Mediate the Protective Effect of Apigenin against Cyclophosphamide Hepatotoxicity

**DOI:** 10.3390/metabo12070648

**Published:** 2022-07-14

**Authors:** Wesam Al-Amarat, Mohammad H. Abukhalil, Reem S. Alruhaimi, Haifa A. Alqhtani, Nouf Aldawood, Manal A. Alfwuaires, Osama Y. Althunibat, Saleem H. Aladaileh, Abdulmohsen I. Algefare, Abdulkareem A. Alanezi, Ali M. AbouEl-ezz, Ahmad F. Ahmeda, Ayman M. Mahmoud

**Affiliations:** 1 Department of Medical Support, Al-karak University College, Al-Balqa’ Applied University, As-Salt 206, Jordan; wsam.amarat@bau.edu.jo; 2 Department of Medical Analysis, Princess Aisha Bint Al-Hussein College of Nursing and Health Sciences, Al-Hussein Bin Talal University, Ma’an 71111, Jordan; mabukhalil@ahu.edu.jo (M.H.A.); osama.y.althunibat@ahu.edu.jo (O.Y.A.); shmoud@uhb.edu.sa (S.H.A.); 3 Department of Biology, College of Science, Al-Hussein Bin Talal University, Ma’an 71111, Jordan; 4 Department of Biology, College of Science, Princess Nourah bint Abdulrahman University, Riyadh 11671, Saudi Arabia; hsalrhemi@pnu.edu.sa (R.S.A.); haalqhtani@pnu.edu.sa (H.A.A.); noaraldawood@pnu.edu.sa (N.A.); 5 Department of Biological Sciences, Faculty of Science, King Faisal University, Al-Ahsa 31982, Saudi Arabia; malfwuaires@kfu.edu.sa (M.A.A.); aalgefare@kfu.edu.sa (A.I.A.); 6 Department of Pharmacy Practice, College of Pharmacy, University of Hafr Al-Batin, Hafr Al-Batin 31991, Saudi Arabia; 7 Department of Pharmaceutics, College of Pharmacy, University of Hafr Al-Batin, Hafr Al-Batin 31991, Saudi Arabia; aalanezi@uhb.edu.sa; 8 Department of Molecular Biology, Faculty of Science, New Mansoura University, Mansoura 35511, Egypt; ali.maged@nmu.edu.eg; 9 Department of Basic Medical Sciences, College of Medicine, Ajman University, Ajman 346, United Arab Emirates; a.ahmeda@ajman.ac.ae; 10 Center of Medical and Bio-allied Health Sciences Research, Ajman University, Ajman 346, United Arab Emirates; 11 Physiology Division, Zoology Department, Faculty of Science, Beni-Suef University, Beni-Suef 62514, Egypt; 12 Department of Life Sciences, Faculty of Science and Engineering, Manchester Metropolitan University, Manchester M1 5GD, UK

**Keywords:** chemotherapy, liver injury, oxidative stress, flavonoids, Nrf2

## Abstract

Liver injury is among the adverse effects of the chemotherapeutic agent cyclophosphamide (CP). This study investigated the protective role of the flavone apigenin (API) against CP-induced liver damage, pointing to the involvement of Nrf2/HO-1 signaling. Rats were treated with API (20 and 40 mg/kg) for 15 days and received CP (150 mg/kg) on day 16. CP caused liver damage manifested by an elevation of transaminases, alkaline phosphatase (ALP), and lactate dehydrogenase (LDH), and histological alterations, including granular vacuolation, mononuclear cell infiltration, and hydropic changes. Hepatic reactive oxygen species (ROS), malondialdehyde (MDA), and nitric oxide (NO) were increased and glutathione (GSH) and antioxidant enzymes were decreased in CP-administered rats. CP upregulated the inflammatory markers NF-κB p65, TNF-α, IL-6, and iNOS, along with the pro-apoptotic Bax and caspase-3. Pre-treatment with API ameliorated circulating transaminases, ALP, and LDH, and prevented histopathological changes in CP-intoxicated rats. API suppressed ROS, MDA, NO, NF-κB p65, iNOS, inflammatory cytokines, oxidative DNA damage, Bax, and caspase-3 in CP-intoxicated rats. In addition, API enhanced hepatic antioxidants and Bcl-2 and boosted the Nrf2 and HO-1 mRNA abundance and protein. In conclusion, API is effective in preventing CP hepatotoxicity by attenuating oxidative stress, the inflammatory response, and apoptosis. The hepatoprotective efficacy of API was associated with the upregulation of Nrf2/HO-1 signaling.

## 1. Introduction

Cyclophosphamide (CP) is an alkylating chemotherapeutic agent broadly employed in the therapy of numerous human malignancies [1,2,3]. CP has been approved by the FDA for use in the treatment of stage III and IV lymphomas, including lymphocytic Hodgkin and non-Hodgkin lymphomas [4,5]. CP is used as an immunosuppressant to prevent post-transplant rejection and graft-vs.-host complications; however, its therapeutic application is associated with serious adverse effects, such as hemorrhagic cystitis, myelosuppression, kidney injury, amenorrhea, lung injury, cardiotoxicity, and hepatotoxicity [1,3,6,7,8,9,10,11,12,13]. The metabolism of CP within the liver through the action of cytochrome P-450 produces acrolein and phosphoramide mustard. Acrolein has no antitumor efficacy while phosphoramide mustard is responsible for the majority of the antineoplastic activity of CP [2]. Phosphoramide mustard induces apoptosis via irreversible inhibition of protein synthesis through DNA cross-linking [2]. Acrolein is a highly reactive metabolite with a short half-life and can damage cellular macromolecules [14]. Acrolein can induce toxicity via various direct and indirect mechanisms, including DNA and protein adduction, excessive reactive oxygen species (ROS) generation, and induction of mitochondrial and endoplasmic reticulum (ER) stresses [14]. Excess ROS can damage cellular macromolecules, resulting in lipid peroxidation (LPO) and oxidative DNA and protein damage [15,16]. In addition, ROS can elicit proinflammatory responses via the activation of nuclear factor-kappaB (NF-κB) and the release of proinflammatory mediators [17,18]. Excess ROS and proinflammatory cytokines work in concert to induce cell death via apoptosis [19,20]. Therefore, attenuation of surplus ROS and cytokine production can protect hepatocytes against CP toxicity.

**Figure 1:**
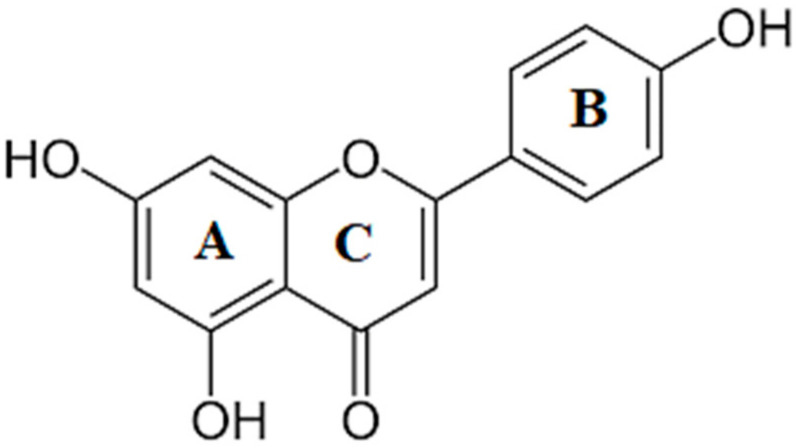
Chemical structure of apigenin.

Apigenin (API; 4,5,7-trihydroxyflavone, Figure 1) is a member of the flavone class that is naturally abundant in various fruits and vegetables. Since ancient times, API has been wieldy applied in traditional medicine because of its antioxidant, hepatoprotective, antiviral, and anti-inflammatory activities [21]. Many recent reports have also emphasized that API exhibits a protective role against chemically induced hepatic toxicity via boosting the antioxidant and ROS scavenging properties [22,23,24,25]. Although numerous pharmacological activities of API have been explored, its potential to attenuate CP-induced hepatic damage has not been investigated. Therefore, this study evaluated the potential of API to protect against oxidative stress, inflammation, and liver injury induced by CP in rats, pointing to the possible involvement of nuclear factor (erythroid-derived 2)-like 2/heme oxygenase1 (Nrf2/HO-1) signaling. Nrf2 is a cytoprotective transcription factor that controls antioxidant defense enzymes and prevents oxidative damage [26,27,28]. Cytosolic Nrf2 is sequestered by Keap1 under physiological conditions and dissociates and translocates into the nucleus upon exposure to ROS or electrophiles, where it binds the antioxidant response element (ARE) and promotes the transcription of HO-1 and other defensive enzymes [26]. The role of Nrf2 activation in preventing CP-induced liver injury has been previously demonstrated [17,29,30,31,32]. Thus, we hypothesized that upregulation f Nrf2/HO-1 is involved in the protective effect of API against liver injury induced by CP.

## 2. Results

### 2.1. API Prevents CP-Induced Hepatocyte Injury in Rats

**Figure 2:**
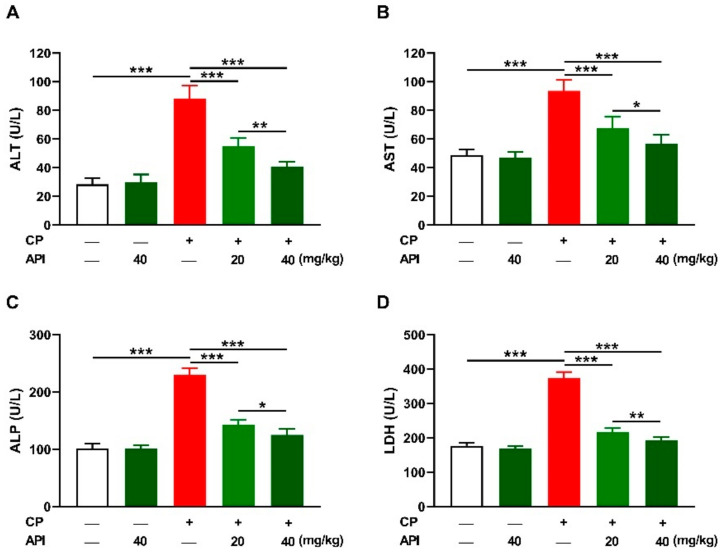
API ameliorated serum (**A**) ALT, (**B**) AST, (**C**) ALP, and (**D**) LDH in CP-intoxicated rats. Data are mean ± SEM, (*n* = 6). * *p* < 0.05, ** *p* < 0.01, and *** *p* < 0.001.

**Figure 3:**
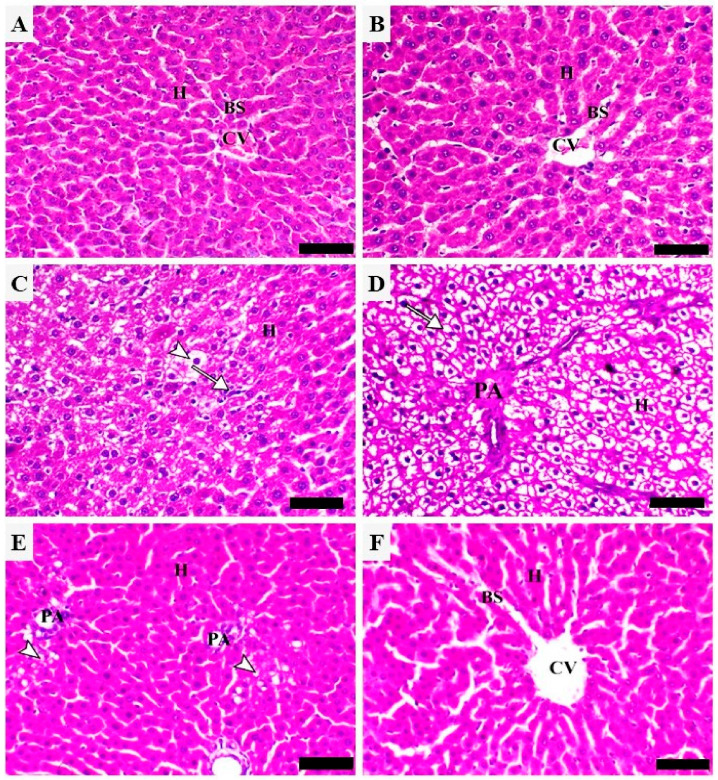
API prevented CP-induced tissue injury. Photomicrographs of sections in the liver of (**A**) control and (**B**) API-administered rats showing a normal histological architecture with normal hepatocytes (H) radially arranged in cords around central veins (CVs) and normal sinusoids (BS); (**C**,**D**) CP-intoxicated rats showing marked granular hepatic vacuolation (arrowheads), mononuclear cells infiltration (arrow) [C], and advanced hydropic changes (arrow) [D], PA indicates portal area and H indicates hepatocytes; (**E**,**F**) CP-intoxicated rats treated with 20 mg/kg API (**E**) showing focally limited periportal hepatic vacuolation (arrowheads), and 40 mg/kg API (**F**) showing a marked decrease in hepatic vacuolation with mild congestion of the blood sinusoids (H&E, Scale bar = 50 µm).

The protective effect of API against hepatocyte damage induced by CP was evaluated via measurement of circulating markers (Figure 2) and microscopic examination of stained sections (Figure 3). CP increased alanine aminotransferase (ALT), aspartate aminotransferase (AST), alkaline phosphatase (ALP), and lactate dehydrogenase (LDH) in the serum of rats (*p* < 0.001) as depicted in Figure 2A–D. API ameliorated transaminases, ALP, and LDH in CP-intoxicated rats in a dose-dependent manner. Rats that received 40 mg/kg API exhibited no changes in liver function markers.

Both control (Figure 3A) and API-treated rats (Figure 3B) showed normal histology of the hepatic tissue, where hepatocytes appeared normal and radially arranged in cords around central veins. CP-intoxicated rats exhibited multiple histopathological changes, including degenerative changes, marked granular hepatic vacuolation, mononuclear cell infiltration, and advanced hydropic changes (Figure 3C,D). Treatment with the low dose of API improved the tissue architecture, which showed focally limited periportal hepatic vacuolation (Figure 3E). The 40 mg/kg API dose markedly decreased hepatic vacuolation and other degenerative changes and the sections revealed mild congestion of the blood sinusoids (Figure 3F).

### 2.2. API Attenuates CP-Induced Oxidative Stress and Oxidative DNA Damage in Rats

**Figure 4:**
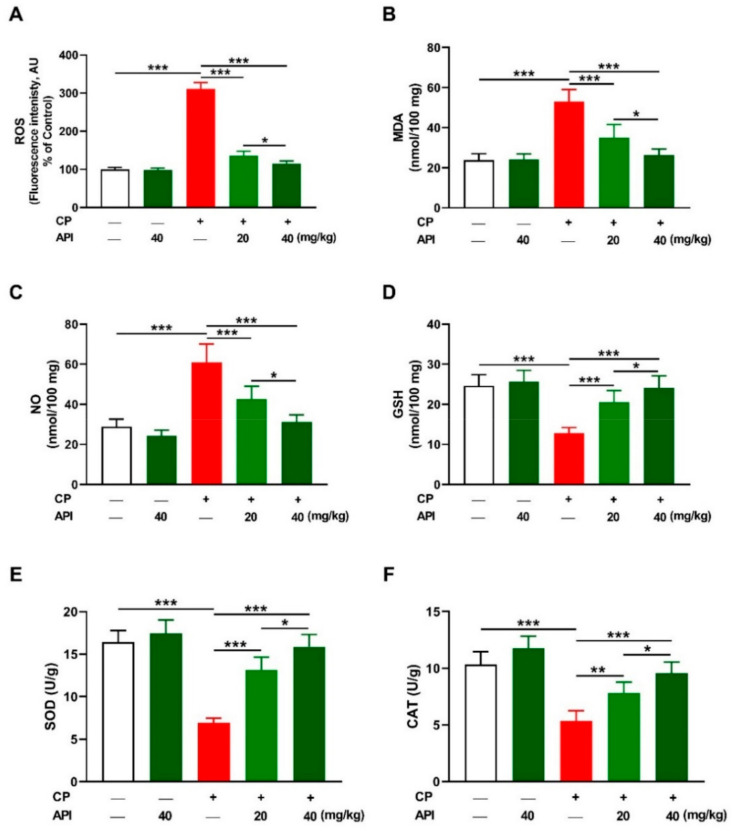
API attenuated CP-induced hepatic oxidative stress. API decreased (**A**) ROS, (**B**) MDA, and (**C**) NO, and increased (**D**) GSH, (**E**) SOD, and (**F**) CAT in the liver of CP-administered rats. Data are mean ± SEM, (*n* = 6). * *p* < 0.05, ** *p* < 0.01, and *** *p* < 0.001.

**Figure 5:**
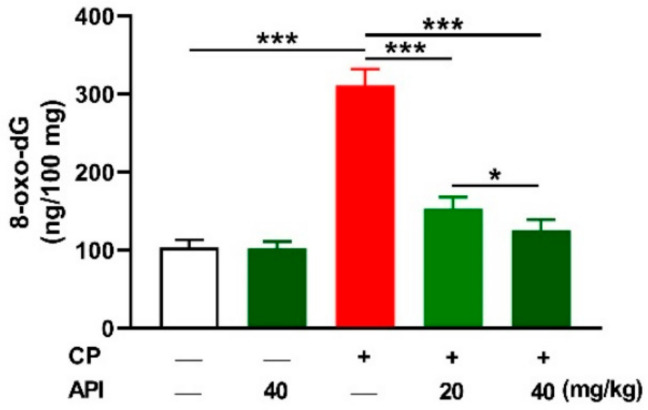
API ameliorated oxidative DNA damage in CP-intoxicated rats. Pre-treatment with API decreased hepatic 8-oxo-dG in a dose-dependent manner. Data are mean ± SEM, (*n* = 6). * *p* < 0.05 and *** *p* < 0.001.

Excessive ROS levels play a key role in CP hepatotoxicity, and the results revealed a significant elevation in the liver of CP-intoxicated rats (Figure 4A) as compared to the control (*p* < 0.001). Hepatic malondialdehyde (MDA; Figure 4B) and nitric oxide (NO; Figure 4C) were markedly elevated in rats that received CP (*p* < 0.001). Treatment with either dose of API decreased hepatic ROS, MDA, and NO significantly (*p* < 0.001) in CP-intoxicated rats. Reduced glutathione (GSH), superoxide dismutase (SOD), and catalase (CAT) were significantly declined in CP-intoxicated rats (*p* < 0.001), an effect that was reversed in the API-treated groups (Figure 4D–F). The effect of API on hepatic oxidants and antioxidants in CP-intoxicated rats was dose-dependent, whereas it had no effect in normal rats. 8-Oxo-2′-deoxyguanosine (8-oxo-dG), a marker of oxidative DNA damage, was significantly increased in the liver of CP-intoxicated rats, an effect that was dose-dependently prevented by API (Figure 5).

### 2.3. API Activates Nrf2/HO-1 Signaling in CP-Intoxicated Rats

**Figure 6:**
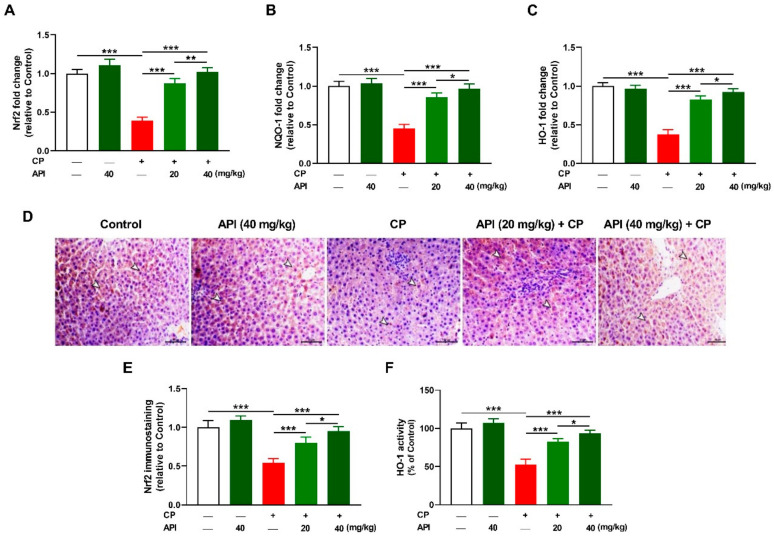
API activated hepatic Nrf2/HO-1 signaling in CP-intoxicated rats. API increased the (**A**) Nrf2, (**B**) NQO-1, and (**C**) HO-1 mRNA abundance, and (**F**) HO-1 activity in CP-administered rats. (**D**) Immunostaining of hepatic Nrf2 (arrowheads) showed a decrease in the expression in CP-administered rats and upregulation in the API-treated groups. (**E**) Image analysis of Nrf2 immunostaining. Data are mean ± SEM, (*n* = 6). * *p* < 0.05, ** *p* < 0.01, and *** *p* < 0.001.

CP downregulated the hepatic Nrf2 (Figure 6A), NAD(P)H quinone dehydrogenase (NQO)-1 (Figure 6B), and HO-1 (Figure 6C) mRNA abundance when compared with the control group (*p* < 0.001). Nrf2 protein (Figure 6D–E) was downregulated and HO-1 activity (Figure 6F) was suppressed in the liver of rats that received CP (*p* < 0.001). API dose-dependently upregulated hepatic Nrf2, NQO-1, and HO-1 in CP-intoxicated rats (*p* < 0.001) while having a non-significant effect on Nrf2/HO-1 signaling in normal rats.

### 2.4. API Mitigates CP-Induced Hepatic Inflammation in Rats

**Figure 7:**
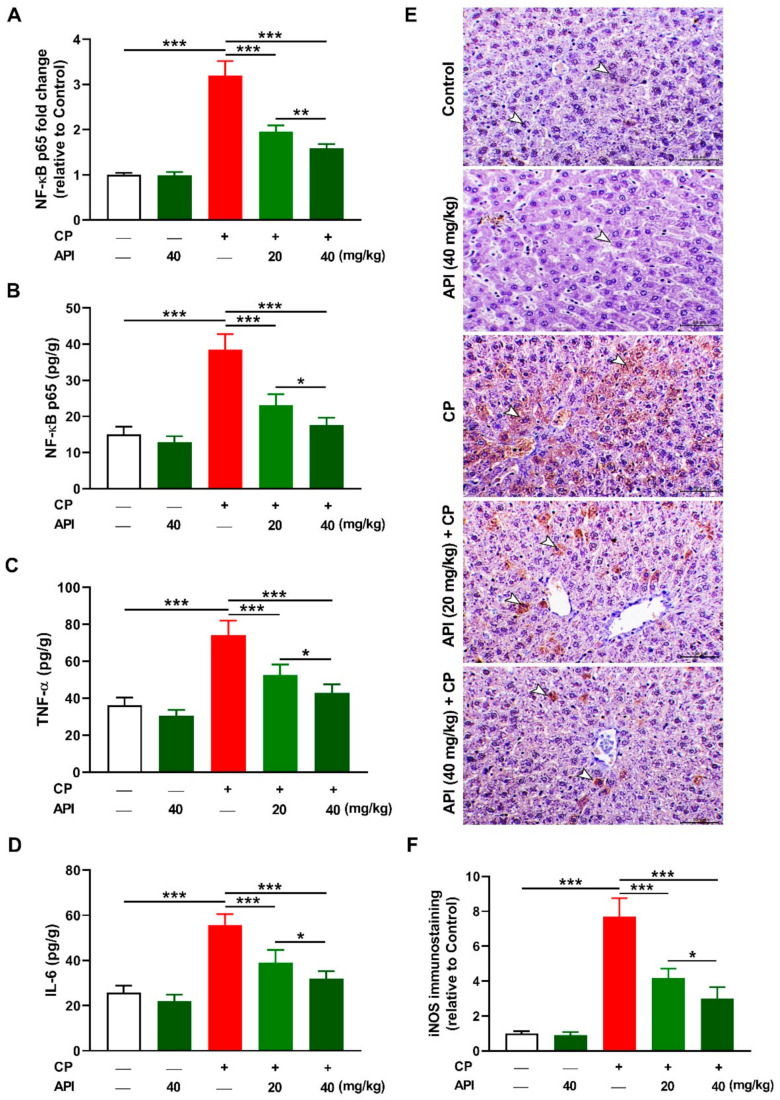
API mitigated inflammation in CP-intoxicated rats. API decreased hepatic (**A**) NF-κB p65 mRNA, (**B**) NF-κB p65 protein, (**C**) TNF-α, and (**D**) IL-6 in CP-administered rats. (**E**,**F**) Photomicrographs showing increased immunostaining of iNOS (**E**) and image analysis (**F**) showing that API decreased hepatic iNOS in CP-administered rats. Data are mean ± SEM, (*n* = 6). * *p* < 0.05, ** *p* < 0.01, and *** *p* < 0.001.

NF-κB, tumor necrosis factor (TNF)-α, interleukin (IL)-6, and inducible NO synthase (iNOS) were measured to evaluate the anti-inflammatory activity of API. CP upregulated hepatic NF-κB p65 mRNA (Figure 7A) and protein (Figure 7B) in CP-intoxicated rats (*p* < 0.001). Similarly, hepatic TNF-α (Figure 7C), IL-6 (Figure 7D), and iNOS (Figure 7E,F) showed a significant increase in CP-administered rats (*p* < 0.001). Treatment with 20 or 40 mg/kg API downregulated NF-κB p65 and the proinflammatory mediators in CP-administered rats, with the higher dose exerting a more potent effect. Normal rats treated with 40 mg/kg API exhibited no changes in the assayed inflammatory markers.

### 2.5. API Prevents CP-Induced Hepatic Apoptosis in Rats

**Figure 8:**
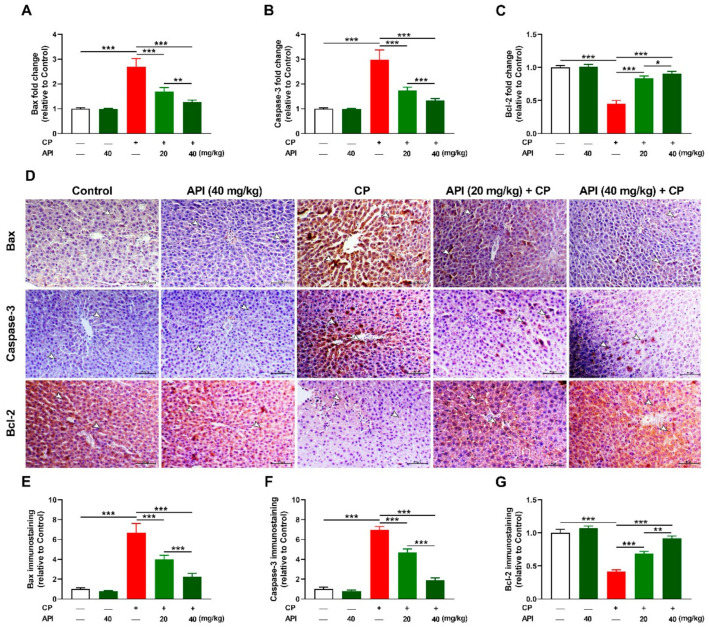
API prevented CP-induced hepatocyte apoptosis in rats. API decreased (**A**) Bax and (**B**) caspase-3 and increased (**C**) Bcl-2 mRNA in CP-administered rats. (**D**) Immunostaining of Bax, caspase-3, and Bcl-2, and (**E**–**G**) image analysis showing decreased (**E**) Bax and (**F**) caspase-3 and increased (**G**) Bcl-2 in CP-intoxicated rats treated with API. Data are mean ± SEM, (*n* = 6). * *p* < 0.05, ** *p* < 0.01, and *** *p* < 0.001.

CP provoked apoptosis in the liver of rats as shown by the significant increase in Bax (Figure 8A,D,E) and caspase-3 (Figure 8B,D,F), and decreased Bcl-2 (Figure 8C,D,G), both RNA and protein levels, as compared to the control group (*p* < 0.001). While it had no effect in normal rats, API dose-dependently decreased hepatic Bax and caspase-3 and boosted Bcl-2 in CP-intoxicated rats.

## 3. Discussion

Hepatotoxicity is one of the main health complications related to the use of anticancer agents [33]. CP is an effective chemotherapeutic agent against various cancers; however, hepatotoxicity and other toxic complications limit its applications. CP causes liver injury at both low [12] and high doses [13], with oxidative stress playing a major role [14,17,31,32,34]. Herein, we investigated the protective efficacy of the antioxidant and anti-inflammatory flavonoid API [22,23,24,25] against oxidative stress, the inflammatory response, and liver injury in CP-intoxicated rats.

CP injection resulted in liver injury manifested by the elevated serum transaminases, ALP, and LDH, along with multiple histopathological changes, including diffuse vacuolation consistent with advanced hydropic changes associated with mononuclear cell infiltration. Elevated transaminases and other enzymes in serum are a sensitive marker of liver injury since their release indicates hepatocyte injury [35]. The findings of this investigation were consistent with previous studies from Mahmoud’s lab showing a substantial increase in circulating liver function markers and liver tissue injury in rats that received a single injection of CP [17,29,31,32,34,36]. Pre-treatment with API exerted a dose-dependent hepatoprotective effect marked by the ameliorated liver function markers in CP-intoxicated rats. API mitigated CP-induced histopathological alterations and improved the histological architecture of the liver, demonstrating a potent hepatoprotective efficacy. The ability of API to protect against different hepatotoxicants has been previously reported in rodents challenged with NDEA, furan, acetaminophen, and methotrexate (MTX) [22,23,24,25]. In these studies, treatment with API was effective in ameliorating serum transaminases and preventing hepatocyte injury [22]. Our findings add support to the hepatoprotective effect of API by showing its ability to prevent CP-induced liver injury.

Owing to the role of oxidative stress in CP hepatotoxicity [14,17,31,32,34], we assumed that the protective efficacy of API was mediated through attenuation of oxidative injury and improvement of cellular antioxidants. CP provoked excessive generation of ROS that is extensively involved in its deleterious effect on the liver [31]. In addition, acrolein produced during its metabolism increases ROS release and can damage membranes via its direct covalent binding with proteins and lipids [14]. Excessive ROS can damage lipids by provoking LPO, proteins, DNA, and other cellular macromolecules, and deplete GSH and other antioxidants, thereby leading to cell death [37]. Accordingly, increased hepatic MDA, NO, and 8-oxo-dG, and decreased antioxidants in conjunction with surplus ROS were observed in CP-administered rats in this study. LPO destroys the cells by disrupting the membrane fluidity and permeability and inactivating the bound proteins [37]. NO, produced by the upregulated iNOS, can produce the versatile oxidant peroxynitrite (ONOO⁻) by reacting with superoxide radicals. ONOO⁻ can increase ROS production, impair mitochondrial function, and modify DNA bases, leading to DNA breaks and cell injury [38]. This study supports our previous studies showing elevated hepatic ROS, LPO, NO, and oxidative DNA damage along with declined antioxidant defenses in rats that received CP [17,29,31,32,36]. API significantly suppressed hepatic ROS, LPO, NO, and oxidative DNA damage and boosted GSH, SOD, and CAT in CP-intoxicated rats, highlighting its antioxidant efficacy. In this context, API ameliorated LPO and improved GSH and antioxidant enzymes in the liver of rodents intoxicated with NDEA [22], furan [23], acetaminophen [24], and MTX [25]. API possesses a radical scavenging property mediated by the hydrogen atom transfer and single electron transfer pathways [39]. The OH group in its B ring enables API to stabilize free radicals by donating its H and electron, producing a stable flavonoid radical [39]. The 4-oxo group in the C ring and the *m*-5,7-dihydroxy arrangements in the A ring participate in the radical scavenging property of API [39]. Electron delocalization from the B ring to the 4 keto group and the C2-C3 double bond helps API to scavenge free radicals [40]. The antioxidant activity of API could also be connected to its interaction and modulation with cell signaling molecules. We assumed that activation of Nrf2 plays a role in the antioxidant and hepatoprotective efficacy of API. In this study, CP suppressed hepatic Nrf2/HO-1 signaling as evidenced by the decreased mRNA abundance of Nrf2, NQO-1, and HO-1, Nrf2 protein, and HO-1 activity, demonstrating the surplus production of ROS. Although activated by ROS under physiological conditions, Nrf2 downregulation has been reported under conditions of excessive and prolonged ROS, including CP hepatotoxicity [30,31,41,42,43,44]. API upregulated hepatic Nrf2/HO-1 signaling in CP-intoxicated rats as shown by the upregulated Nrf2, NQO-1, and HO-1, which explained the enhanced antioxidant enzymes. Previous investigators have reported the role of Nrf2 in mediating the therapeutic actions of API. This flavone activated Nrf2-mediated expression of antioxidant enzymes and induced anti-inflammatory effects in HepG2 cells [45], and protected PC12 cells [46] and melanocytes against the deleterious effects of ROS by activating Nrf2 signaling [47]. In addition, API activated Nrf2 and protected the liver against oxidative stress in a rodent model of NAFLD [48].

In addition to oxidative stress, API attenuated the hepatic inflammatory response and apoptosis in CP-intoxicated rats. NF-κB, iNOS, TNF-α, and IL-6 were increased in the liver of CP-intoxicated rats. Sustained and surplus ROS activates NF-κB, which plays a key role in inflammation by increasing the transcription of iNOS, TNF-α, IL-6, and other mediators [49]. In liver diseases, the proinflammatory mediators recruit inflammatory cells and provoke the production of more ROS, leading to cell death [50]. Upregulation of NF-κB and increased proinflammatory mediators and inflammatory cell infiltration were associated with surplus ROS levels in the liver of CP-intoxicated rats [17,29,31,51]. Free radicals and proinflammatory mediators work in concert to elicit cell death via apoptosis by activating Bax, which promotes the loss of mitochondrial membrane potential [52]. Subsequently, cytochrome c is released to the cytoplasm, where it activates the executioner caspase-3 [52], which provokes DNA fragmentation, degradation of cytoskeletal proteins, and further release of cytochrome c, leading to cell death [53]. Here, CP increased both gene and protein expression of Bax and caspase-3 and decreased Bcl-2. Similar findings were observed in the liver of rats that received CP [17,29,31]. API suppressed hepatic NF-κB, iNOS, cytokines, Bax, and caspase-3 and upregulated Bcl-2 in CP-intoxicated rats, demonstrating its anti-inflammatory and anti-apoptosis efficacies. Accordingly, API decreased proinflammatory cytokines in the serum of rodent models of furan- [23] and MTX-induced hepatotoxicity [25], and decreased Bax, and increased Bcl-2 in a model of ischemia/reperfusion-induced liver injury [54]. Similar to its antioxidant property, the anti-inflammatory effect of API could be linked to the activation of Nrf2/HO-1 signaling, which prevents NF-ĸB activation and the release of proinflammatory mediators [27,28].

In conclusion, this study introduced new information that the flavone API can protect the liver against CP toxicity by activating Nrf2 signaling and attenuating oxidative injury and the inflammatory response. API alleviated liver function markers, prevented hepatocyte injury, and suppressed ROS, LPO, NF-κB, proinflammatory mediators, and apoptosis markers. These beneficial effects were associated with activation of Nrf2/HO-1 signaling and enhancement of antioxidant defenses. Therefore, API could be a potent protective agent against CP hepatotoxicity in clinical settings, pending further investigations and clinical trials to explore its exact therapeutic mechanism(s).

## 4. Materials and Methods

### 4.1. Experimental Design and Treatments

Thirty male Wistar rats (200–220 g) were housed under standard conditions on a 12 h light/dark cycle and provided with free access to food and water. The rats were acclimatized for 7 days and then allocated into 5 groups (*n* = 6). Group I served as a control and received the vehicle 0.5% carboxymethyl cellulose (CMC, Sigma, St. Louis, MO, USA), and group II received 40 mg/kg API (Sigma, St. Louis, MO, USA) suspended in 0.5% CMC [55] via oral gavage for 15 days. Groups III, IV, and V received a single intraperitoneal injection of 150 mg/kg CP (Endoxan^®^, Baxter Oncology, Halle, Germany) diluted in sterile saline on day 16 [29,31]. Group III received 0.5% CMC and groups IV and V received 20 and 40 mg/kg API in 0.5% CMC, respectively, via oral gavage for 15 days [55].

On the 19th day, blood was collected via cardiac puncture under ketamine/xylazine anesthesia and serum was prepared by centrifuging the samples at 3000 for 15 min. The rats were sacrificed and dissected to excise the livers of specimens, which were fixed in 10% neutral buffered formalin (NBF) for histology and immunohistochemistry. Specimens from the liver were stored in RNAlater at −80 °C for RNA isolation while other specimens were homogenized (10% *w*/*v*) in cold Tris-HCl buffer (pH = 7.4), centrifuged, and the clear supernatant was kept at −80 °C for biochemical analyses.

### 4.2. Determination of Liver Function Enzymes, Cytokines, and 8-Oxo-dG

The activities of transaminases (ALT and AST), ALP, and LDH were assayed in the serum of rats using reagent kits supplied by Spinreact (Girona, Spain). TNF-α and IL-6 levels were measured in liver homogenates using R&D Systems (Minneapolis, MN, USA) ELISA kits and NF-κB p65 was determined using an ELISA kit (Elabscience, Houston, TX, USA). 8-Oxo-dG was assayed by an ELISA kit supplied by Cusabio (Wuhan, China). All assays were conducted following the manufacturers’ instructions.

### 4.3. Determination of ROS, LPO, NO, and Antioxidants

Hepatic ROS levels were assayed by mixing 100 μL sample with 5 μL H_2_DCF-DA (Sigma, St. Louis, MO, USA) in 1 mL PBS and incubation of the mixture at 37 °C protected from the light. After 30 min, the fluorescence was determined at 490 nm [56]. MDA, a marker of LPO, was measured as described by Ohkawa et al. [57], and NO was assayed using Griess reagent [58]. GSH, SOD, and CAT were assayed following the methods described by Ellman [59], Marklund and Marklund [60], and Cohen et al. [61], respectively. Total HO-1 activity was assayed as reported by Abraham et al. [62].

### 4.4. Histopathology and Immunohistochemistry (IHC) Examination

Liver samples fixed in 10% NBF for 24 h were processed for paraffin embedding and 5-µm sections were cut using a microtome. The sections were stained with hematoxylin and eosin (H&E) as previously described [63]. To evaluate changes in the expression levels of iNOS, Bax, Bcl-2, caspase-3, and Nrf2, sections from the liver were dewaxed and 0.05 M citrate buffer (pH 6.8) was used for antigen retrieval. The slides were treated with 0.3% hydrogen peroxide (H_2_O_2_), washed in phosphate-buffered saline (PBS), and probed with anti-iNOS (ThermoFisher Scientific, Waltham, MA, USA; 1:20 dilution), anti-Bax (Abcam, Cambridge, MA, USA; 1:100 dilution), anti-Bcl-2 (Abcam, Cambridge, UK; 1:100 dilution), anti-caspase-3 (ThermoFisher Scientific, Waltham, MA, USA; 1:100 dilution), and anti-Nrf2 (ThermoFisher Scientific, Waltham, MA, USA; 1:100 dilution) overnight at 4 °C. The slides were washed in PBS, incubated with secondary antibodies, and color was developed by incubation with DAB in H_2_O_2_ [64]. Mayer’s hematoxylin was used for counterstaining, and the slides were visualized under a light microscope. The intensity of the developed color was determined in 6 fields/slide using ImageJ (NIH, Bethesda, MD, USA).

### 4.5. qRT-PCR

The effect of CP and API on hepatic Nrf2, HO-1, NQO-1, Bax, Bcl-2, and caspase-3 was evaluated using qRT-PCR as previously described [65]. TRIzol (ThermoFisher Scientific, Waltham, MA, USA) was used to isolate RNA, which was treated with RNase-free Dnase (Qiagen, Germany), and its quantity and purity were determined using a nanodrop. RNA samples with A260/A280 of 1.8 or more were used for reverse transcription using a cDNA synthesis kit (ThermoFisher Scientific, Waltham, MA, USA). Amplification of the cDNA was carried out by SYBR Green (ThermoFisher Scientific, Waltham, MA, USA) and the primers in Table 1. The obtained data were analyzed using the 2^−ΔΔCt^ method [66] and normalized to β-actin.

### 4.6. Statistical Analysis

The results are expressed as mean ± standard error of the mean (SEM) and the statistical comparisons were carried out by one-way ANOVA and Tukey’s test n GraphPad Prism 8 software (GraphPad, San Diego, CA, USA). A *p* value < 0.05 was considered significant.

## Figures and Tables

**Table 1:** Primers used for qRT-PCR.

Gene	Forward Primer (5′-3′)	Reverse Primer (5′-3′)
Nrf2	TTGTAGATGACCATGAGTCGC	TGTCCTGCTGTATGCTGCTT
NQO-1	GGCCATCATTTGGGCAAGTC	TCCTTGTGGAACAAAGGCGA
HO-1	GTAAATGCAGTGTTGGCCCC	ATGTGCCAGGCATCTCCTTC
NF-κB p65	CCTCATCTTTCCCTCAGAGCC	GGTCCCGTGTAGCCATTGAT
Caspase-3	GGAGCTTGGAACGCGAAGAA	ACACAAGCCCATTTCAGGGT
Bax	AGGACGCATCCACCAAGAAG	CAGTTGAAGTTGCCGTCTGC
Bcl-2	ACTCTTCAGGGATGGGGTGA	TGACATCTCCCTGTTGACGC
β-actin	AGGAGTACGATGAGTCCGGC	CGCAGCTCAGTAACAGTCCG

## Data Availability

The data presented in this study are available in article.
